# YTHDF1 upregulation mediates hypoxia-dependent breast cancer growth and metastasis through regulating PKM2 to affect glycolysis

**DOI:** 10.1038/s41419-022-04711-1

**Published:** 2022-03-23

**Authors:** Xuemei Yao, Wei Li, Liqi Li, Menghuan Li, Youbo Zhao, De Fang, Xiaohua Zeng, Zhong Luo

**Affiliations:** 1 School of Life Science, Chongqing University, Chongqing, 400044 China; 2 Chongqing Key Laboratory of Translational Research for Cancer Metastasis and Individualized Treatment, Chongqing University Cancer Hospital, Chongqing, 400030 China; 3 Department of General Surgery, Xinqiao Hospital, Army Medical University, Chongqing, 400037 China; 4 Center for Tissue Engineering and Stem Cell Research, National Joint Local Engineering Laboratory for Cell Engineering and Biomedicine Technique, Guizhou Medical University, Guiyang, 550004 China

**Keywords:** Breast cancer, Oncogenes

## Abstract

N6-methyladenosine modification is the most common RNA modification mechanism in mammals. YTHDF1, a m^6^A reader, can recognize the m^6^A of mRNAs to facilitate the interaction with the mRNA ribosome assembly and recruitment of translation initiators to promote translation. From a clinical perspective, YTHDF1 upregulation is frequently observed in breast cancer, but its involvement in those cancer-related events is still unclear. Here we report that YTHDF1 is a cancer driver capable of facilitating the proliferation and invasion of breast cancer cells as well as enhancing tumorigenicity and metastasis through promoting glycolysis. We found that tumor hypoxia can transcriptionally induce HIF1α and post-transcriptionally inhibit the expression of miR-16-5p to promote YTHDF1 expression, which could sequentially enhance tumor glycolysis by upregulating PKM2 and eventually increase the tumorigenesis and metastasis potential of breast cancer cells. Inhibiting YTHDF1 via gene knockdown or miR-16-5p would significantly abolish YTHDF1-dependent tumor growth and metastasis. In summary, we identified the role of the YTHDF1-PKM2 signal axis in the occurrence and development of breast cancer, which can be used as a potential target for breast cancer treatment.

## Introduction

Breast cancer is one of the leading causes of cancer-related deaths among women around the globe and its morbidity and mortality rates are increasing rapidly [1]. However, despite the tremendous progress in the diagnosis and targeted treatment for breast cancer in recent years, the survival of breast cancer patients is still not satisfactory [2–6]. Recent investigations collectively demonstrated that the treatment failure against breast cancer is primarily associated with the hypoxic microenvironment, which not only confers resistance to various conventional therapeutic modalities but also enhances their metastatic potential [7–9]. For instance, it’s well established that hypoxia could activate multiple damage-repair mechanisms and signaling pathways to escape the cytotoxic treatment [10–12]. Alternatively, hypoxia may also stimulate the glycolysis metabolism of tumor cells to facilitate energy production and promote tumor metastasis [13–15]. Consequently, it would be of clinical significance to identify new molecular biomarkers as therapeutic targets to circumvent the intrinsic resistance of breast cancer to conventional chemotherapy as well as to ameliorate the risk of metastasis.

m^6^A methylation modification is the most abundant modification in all RNAs, which accounts for 20–40% of total RNA modification events in mammals and is also responsible for approximately 40% of all methylated ribonucleosides in cellular RNA and 0.1–0.4% of the total adenosine [16–18]. m^6^A modification affects almost every stage of RNA metabolism including splicing, maturation, export, translation, and decay, and is an important regulator of mammalian gene expression [19–21]. Emerging evidence shows that the elevated m^6^A modification and the dysregulated expression of related proteins are closely related to the occurrence and development of a variety of human diseases, especially cancer [22–24]. For instance, several cancer indications such as acute myeloid leukemia may demonstrate abnormal levels of m^6^A modification, while inhibiting the activity or expression of m^6^A modification-related enzymes could suppress the malignant progression [25–28]. Recently, YTH N6-methyladenosine RNA binding protein 1 (YTHDF1), a member of the YTH domain family in the cytoplasm, has been suggested to play an important role in protein translation by recruiting transcription initiators to target mRNA. Clinical evidence confirms that YTHDF1 is commonly up-regulated in many cancers and could maintain hypoxia tolerance by promoting the translation of certain proteins [29–32]. Nevertheless, the role of YTHDF1-medaited m^6^A modification in the hypoxia-driven emergence, development, and metastasis of breast cancer cells remains largely unknown, which hampers the potential therapeutic exploitation of the associated mechanisms.

Herein, we demonstrate that the YTHDF1 upregulation is a driving factor in breast cancer cells, which could enhance the proliferation potential and invasiveness of breast cancer cells by promoting protein translation of PKM2 to enhance glycolysis. YTHDF1 knockdown can significantly inhibit breast cancer cell proliferation, colony formation, invasion, and enhance cell apoptosis, leading to substantially enhanced antitumor efficacy. Meanwhile, we also identify that YTHDF1 is involved in the hypoxia-enabled tumor promotion effect, for which the hypoxic tumor microenvironment can inhibit the expression of endogenous miR-16-5p and up-regulate the expression of YTHDF1. In addition, we determined that PKM2 is a target gene that could activate YTHDF1 to facilitate the occurrence and development of breast cancer. Based on the experimental results, we revealed that the YTHDF1-mediated m6A methylation modification could be a promising target for the development of more advanced breast cancer therapies with enhanced efficacy and selectivity.

## Results

### Functional analysis identifies YTHDF1 as a pro-tumorigenic driver in breast cancer

**Fig. 1:**
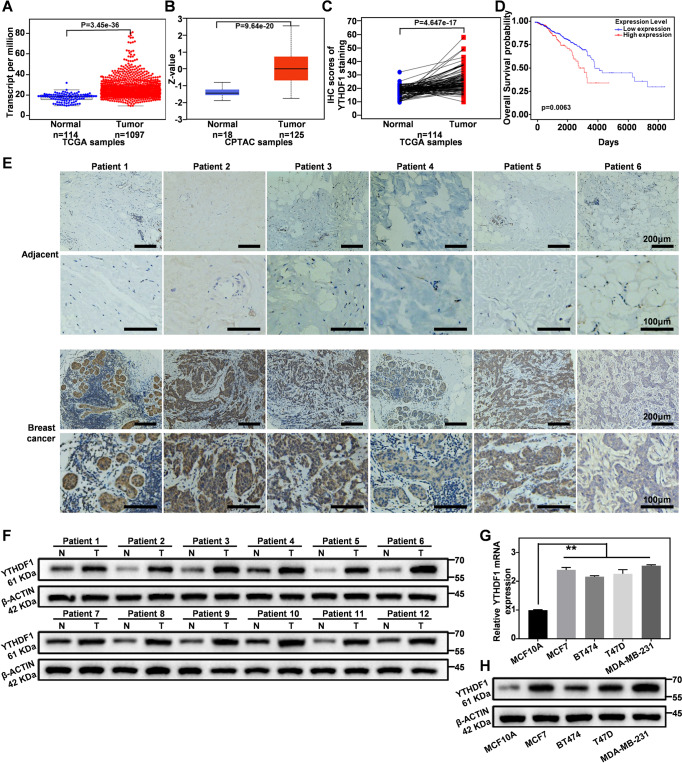
YTHDF1 is up-regulated in breast cancer and correlated with poor prognosis. **A**, **B** Relative mRNA and protein expression levels of YTHDF1 in breast cancer based on TCGA and CPTAC datasets, respectively. **C** YTHDF1 immunohistochemical staining of matched normal breast tissue and breast cancer tissue in TCGA dataset. **D** Kaplan–Meier analysis of the correlation between YTHDF1 expression and overall survival in breast cancer patients based on TCGA dataset. **E** Representative immunohistochemical images of YTHDF1 in patient-derived breast cancer and normal breast tissues. **F** Protein expression of YTHDF1 in 12 pairs of patient-derived breast cancer and normal breast tissues via western blotting. **G**, **H** RT-PCR and western blot analysis of the expression levels of YTHDF1 in normal breast cell line and breast cancer cell lines. Statistical analysis results are presented as mean ± SEM, student’s t test, *P < 0.05, ** *P* < 0.01, ***P < 0.001.

To investigate the role of YTHDF1 in the development of human breast cancer, we first analyzed the expression of YTHDF1 in breast cancer at transcriptome and protein expression levels using The Cancer Genome Atlas (TCGA) dataset and Clinical Proteomic Tumor Analysis Consortium (CPTAC). Specifically, differential expression analysis showed that the mRNA and protein levels of YTHDF1 in breast cancer tissues were significantly higher than the adjacent normal tissues (Fig. 1A, B), which is also supported by the immunohistochemical results of tissue samples from 114 matched breast cancer patients according to the TCGA database (Fig. 1C). Meanwhile, it was found that high YTHDF1 expression was usually associated with reduced survival in patients with breast cancer based on Gene Expression Profiling Interactive Analysis 2 (GEPIA2) (Fig. 1D). In addition, we collected matched cancer and paracancerous tissues from 22 breast cancer patients for immunohistochemical analysis. The results showed that the cancer tissues of 18 samples showed different degrees of elevated expression of YTHDF1 compared with the paracancerous tissues, while the expression level of YTHDF1 in 4 cases was not significantly higher or even lower than that in adjacent tissues (Fig. 1E and Supplementary Fig. S1). The result showed that the expression trend of YTHDF1 was consistent with the immunohistochemical data of TCGA. We also analyzed the expression of YTHDF1 in 12 matched pairs of breast cancer and adjacent normal tissues from real-life patients via western blot, of which the results consistently revealed that YTHDF1 was substantially upregulated in breast cancer tissues (Fig. 1F; Original western blot data 1). Furthermore, we have also comparatively analyzed the mRNA and protein levels of YTHDF1 in MCF 10A (a human normal breast epithelial cell line) and multiple breast cancer cell lines and observed similar trends (Fig. 1G, H; Original western blot data 2). These findings collectively demonstrate that YTHDF1 upregulation is a hallmark of breast cancers and plays a pro-tumorigenic role in their development and progression.

### YTHDF1 regulates proliferation, invasion, and apoptosis of breast cancer cells

**Fig. 2:**
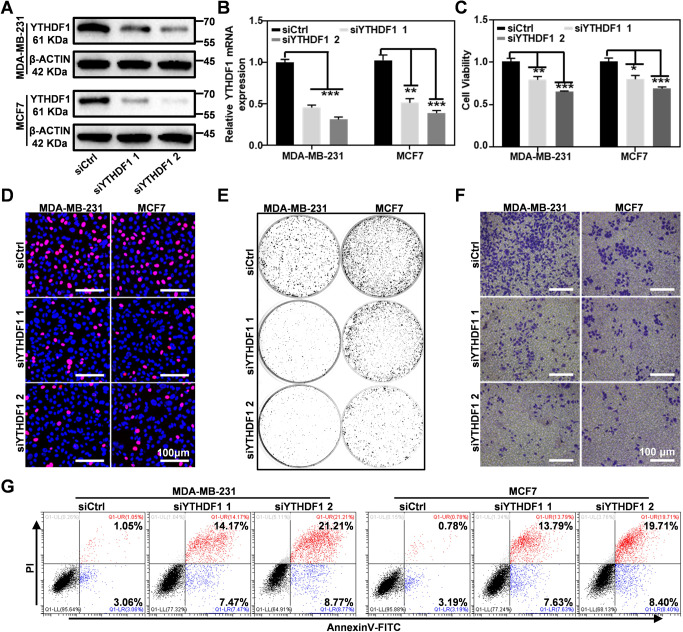
YTHDF1 knockdown inhibited cell growth and invasion in vitro. **A** and **B** Western blot and RT-PCR analysis for YTHDF1 expression in MDA-MB-231 and MCF7 cells transfected with YTHDF1 -inhibiting siRNAs. **C** CCK8 assays on the proliferation of MDA-MB-231 and MCF7 cells after YTHDF1 knockdown. **D** EdU assays of MDA-MB-231 and MCF7 cells after YTHDF1 knock-down. **E** Colony formation assays of MDA-MB-231 and MCF7 cells after YTHDF1 knockdown. **F** YTHDF1 knockdown decreased the invasive potential of MDA-MB-231 and MCF7 cells. **G** FACS analysis on the apoptosis of MDA-MB-231 and MCF7 cells after YTHDF1 knockdown. Statistical analysis results are presented as mean ± SEM, student’s t test, *P < 0.05, ** *P* < 0.01, ***P < 0.001.

To further elucidate the potential regulatory role of YTHDF1 in breast cancer, we genetically depleted YTHDF1 in breast cancer cells and systematically monitored the phenotypical changes after the transfection of two YTHDF1-targeting siRNAs (siYTHDF1 1 and siYTHDF1 2). Typically, the mRNA and protein expression levels for YTHDF1 have decreased significantly after the transfection of both siRNAs, indicating the efficient YTHDF1 knockdown thereof (Fig. 2A, B; Original western blot data 3). CCK8 assay showed that the siRNA-mediated YTHDF1 knockdown significantly inhibited the growth of MDA-MB-231 and MCF7 cells (Fig. 2C). We further verified the suppressing effect of YTHDF1 knockdown on breast cancer cell proliferation by EDU staining (Fig. 2D and Supplementary Fig. S2A, D), in which the cell proliferation of the YTHDF1 siRNA group has been reduced by about 20%. In addition to the proliferation analysis, it was found that the YTHDF1 knockdown could also suppress the colony formation of breast cancer cells (Fig. 2E and Supplementary Fig. S2B). Meanwhile, cell migration and invasion experiment showed that YTHDF1 depletion substantially impaired the migration and invasion capabilities of MDA-MB-231 and MCF7 cells (Fig. 2F and Supplementary Fig. S2C, E). The impact on YTHDF1 knockdown on the malignancy of breast cancer cells was further quantitatively examined by flow cytometry with Annexin V/PI staining, of which the results demonstrated YTHDF1 knockdown caused pronounced apoptosis in MDA-MB-231 and MCF7 with an apoptotic cell population of 29.98 and 28.11%, respectively (Fig. 2G). In addition, we also detected the viability and apoptosis of MCF10A cells transfected with YTHDF1 siRNA by CCK8 and flow cytometry, respectively. The results showed that siYTHDF1 transfection had no significant effect on the vitality of healthy MCF10A cells with negligible changes in cell viability and apoptosis rate (Fig. S2F, G), indicating that YTHDF1 inhibition had no significant effect on normal breast cells and supporting the safety of YTHDF1 inhibition as an antitumor modality in a clinical context. In short, our results indicate that YTHDF1 plays an important role in the maintenance of malignancy of breast cancer cells.

### Hypoxia drives YTHDF1 up-regulation in breast cancer cells

**Fig. 3:**
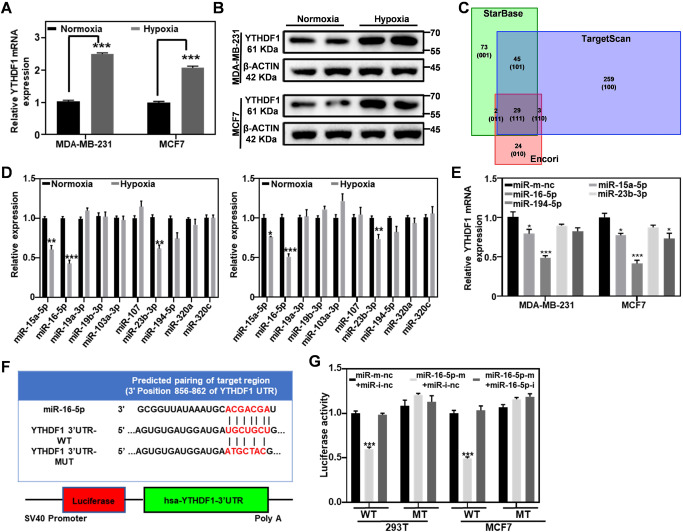
Hypoxia induces YTHDF1 expression in breast cancer cells. **A**, **B** RT-PCR and western blot analysis on the YTHDF1 expression in MDA-MB-231 and MCF7 cells under normoxia and hypoxia. **C** High-throughput screening of TargetScan, Encori, and StarBase for microRNAs targeting the 3′UTR region of YTHDF1 mRNA. **D** RT-PCR analysis on the relative expression of microRNAs in MDA-MB-231 (left) and MCF7 (right) under normoxia and hypoxia. **E** Detection of YTHDF1 mRNA expression levels in breast cancer cell lines after transfection with different microRNAs. **F** The recognition sequence between miRNA-16-5p and YTHDF1 3′UTR, and schematic diagram of the vector construction based on psiCHECK™-2. **G** Co-transfection of miR-16-5p with psiCHECK2.0-YTHDF1 3′UTR fluorescent reporter plasmid on the YTHDF1-specificity of miR-16-5p. Statistical analysis results are presented as mean ± SEM, student’s t test, *P < 0.05, **P < 0.01, ***P < 0.001.

Based on previous clinical reports, hypoxia is a hallmark of many types of breast cancer indications and also a driving factor in their development and progression [33–36]. Typically, hypoxia can induce the expression of multiple key regulators including HIF 1α and HIF 2α, which can activate the transcription of downstream target genes to enhance the oncogenic functions of breast cancer cells [37–40]. To understand the YTHDF1 regulatory mechanism in breast cancer tissues under clinically relevant conditions, we further investigated the impact of hypoxia on the YTHDF1 expression in breast cancer cells. It was found that the mRNA and protein expression levels of YTHDF1 in MDA-MB-231 and MCF7 cell lines were substantially upregulated under hypoxic conditions, suggesting the positive relationship therein (Fig. 3A, B; Original western blot data 4). Furthermore, we transfected HIF1α and HIF2α siRNA into MDA-MB-231 and MCF7 cells and observed that the genetic depletion of HIF1α caused a moderate reduction in YTHDF1 expression, while the depletion of HIF2α had no effect on the expression of YTHDF1 (Supplementary Fig. S3A; Original western blot data 5), indicating that HIF1α is one of the contributing factors in the hypoxia-dependent YTHDF1 regulatory network. The partial YTHDF1 suppression after HIF1α inhibition also shows that there are other regulatory pathways that mediate hypoxia-induced expression of YTHDF1. Alternative to the transcriptional regulation of hypoxia-activatable signaling molecules, it’s well established that hypoxia could mediate the post-transcriptional expression of specific markers by regulating various microRNAs [41, 42]. To investigate the possible involvement of microRNAs in the hypoxia-mediated YTHDF1 expression, we searched across multiple microRNA databases (TargetScan, Starbase, and Encori) to screen 29 microRNAs that might interact with YTHDF1 (Fig. 3C). By referring to the results of the hypoxia-dependent differential expression analysis, we further obtained ten microRNAs (Supplementary Fig. S3B) that were involved in the hypoxia-mediated YTHDF1 overexpression. Subsequently, we systematically investigated the expression of the ten microRNAs under hypoxic conditions and found that the expression levels of miR-16-5p, miR-15a-5p, and miR-23b-3p were significantly down-regulated under hypoxic conditions (Fig. 3D). To study the relevance of these microRNA candidates, we transfected their mimics and found that miR-16-5p mimics showed the greatest inhibition efficacy on the mRNA expression of YTHDF1 (Fig. 3E). Meanwhile, comparative analysis showed that the expression of miR-16-5p in breast cancer cells was evidently lower than that in normal breast epithelial cells (MCF10A) (Supplementary Fig. S3C). Furthermore, we have investigated the expression of miR-16-5p in breast cancer using the microRNA differential expression dataset dbDEMC (https://www.biosino.org/dbDEMC/index) under entry GSE 4589 [43], which showed that the miR-16-5p expression was significantly decreased in breast cancer tissues compared with noncancerous tissues. This is also in good accordance with the findings in another study by Liwei Ruan et al based on the miR-16-5p expression levels in 72 matched breast cancer and adjacent noncancerous tissue pairs. [44] To further elucidate the regulatory role of miR-16-5p in hypoxia-mediated YTHDF1 expression, we constructed YTHDF1 3′UTR wild and mutant dual-luciferase vectors (Fig. 3F and Supplementary Fig. S3D) and co-transfected them with miR-16-5p mimics or inhibitors to detect the luciferase activity. The results showed that miR-16-5p-mediated downregulation of luciferase activity in YTHDF1 3′UTR wild-type could be reversed using miR-16-5p-inhibitor, but not in mutant types where the nucleotides in the seed sequence of the binding site have mutated (Fig. 3G). The above results collectively confirm that the hypoxic tumor microenvironment could upregulate YTHDF1 by enhancing HIF1α expression while inhibiting the expression of endogenous miR-16-5p.

### miR-16-5p targets YTHDF1 to inhibit breast cancer cells

**Fig. 4:**
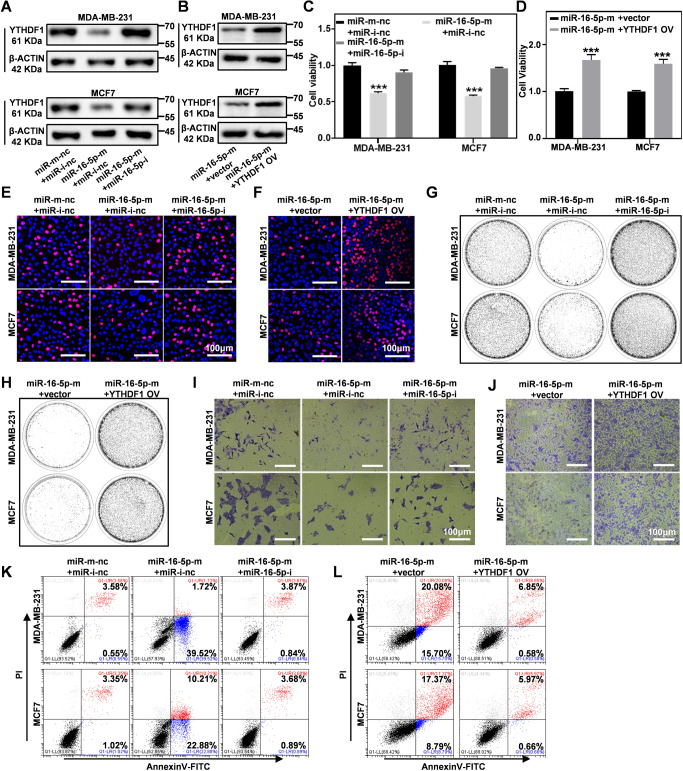
miR-16-5p down-regulates YTHDF1 to inhibit breast cancer cell growth and invasion in vitro. **A**, **B** Western blot analysis for YTHDF1 expression in MDA-MB-231 and MCF7 cells transfected by miR-16-5p mimics/ miR-16-5p inhibitors or co-transfection with miR-16-5p mimics and pCDNA3.1-YTHDF1-3×FLAG. (C and D) CCK8 assays on breast cancer cell growth after the combined treatment of miR-16-5p mimics/miR-16-5p inhibitors or co-transfection with miR-16-5p mimics and pCDNA3.1-YTHDF1-3×FLAG in MDA-MB-231 and MCF7 cells. **E**, **F** EdU assays of MDA-MB-231 and MCF7 cells transfected by miR-16-5p mimics/miR-16-5p inhibitors or co-transfection with miR-16-5p mimics and pCDNA3.1-YTHDF1-3×FLAG. **G**, **H** Colony formation assays of MDA-MB-231 and MCF7 cells transfected by miR-16-5p mimics/miR-16-5p inhibitors or co-transfection with miR-16-5p mimics and pCDNA3.1-YTHDF1-3×FLAG. **I** and **J** miR-16-5p mimics with/without miR-16-5p inhibitors or miR-16-5p mimics with/without pCDNA3.1-YTHDF1-3×FLAG transfection on the invasive abilities of breast cancer cells. **K**, **L** miR-16-5p mimics with/without miR-16-5p inhibitors or miR-16-5p mimics with/without pCDNA3.1-YTHDF1-3×FLAG transfection on the apoptosis levels of MDA-MB-231 and MCF7 cells by FACS. Statistical analysis results are presented as mean ± SEM, student’s t test, *P < 0.05, **P < 0.01, ***P < 0.001.

To determine the impact of the miR-16-5p-mediated YTHDF1 inhibition on breast cancer cells, we systematically monitored the phenotypic changes of breast cancer cells after the transfection of miR-16-5p mimics and the corresponding inhibitors. It was firstly observed that miR-16-5p could abolish the mRNA and protein expression of YTHDF1 in a highly targeted manner. However, co-transfection of miR-16-5p mimics with pCDNA3.1-YTHDF1 × FLAG plasmids restored the protein level of YTHDF1 in breast cancer cells (Fig. 4A, B; Original western blot data 6, 7). Meanwhile, CCK8 assay showed that transfection of miR-16-5p mimics significantly inhibited the proliferation of breast cancer cells, while the combined treatment of miR-16-5p with the treatment with miR-16-5p inhibitor or YTHDF1-expressing plasmids alleviated the miR-16-5p-mediated inhibitory effect (Fig. 4C, D). The cancer inhibitory effect of miR-16-5p and rescuing effect of YTHDF1-expressing plasmids were also consistently supported by EDU staining, which showed similar trends to the results of the CCK analysis (Fig. 4E, F and Supplementary Fig. S4A, B, G, and H). Meanwhile, it was observed that the miR-16-5p treatment also suppressed the cloning, migration and invasive ability of breast cancer cells, while the addition of miR-16-5p inhibitor or pCDNA3.1-YTHDF1×FLAG reversed the tumor-inhibition effect (Fig. 4G–J and Supplementary Fig. S4C, D, I, and J). Flow cytometric analysis based on Annexin V/PI staining further suggested that the transfection of miR-16-5p efficiently induced pronounced apoptosis in MDA-MB-231 and MCF7 cell lines that could be alleviated by YTHDF1-expressing plasmids (Fig. 4K, L). In general, these results collectively demonstrated that miR-16-5p has almost identical YTHDF1-inhibitory functions compared to siRNA-based YTHDF1 knockdown to suppress the malignancy features of breast cancer cells and induce apoptosis, potentiating the application of miR-16-5p as a therapeutic modality to target the YTHDF1 expression in breast cancer cells for efficient tumor inhibition.

### YTHDF1 regulates the expression of PKM2 to promote glycolysis in breast cancer cells

**Fig. 5:**
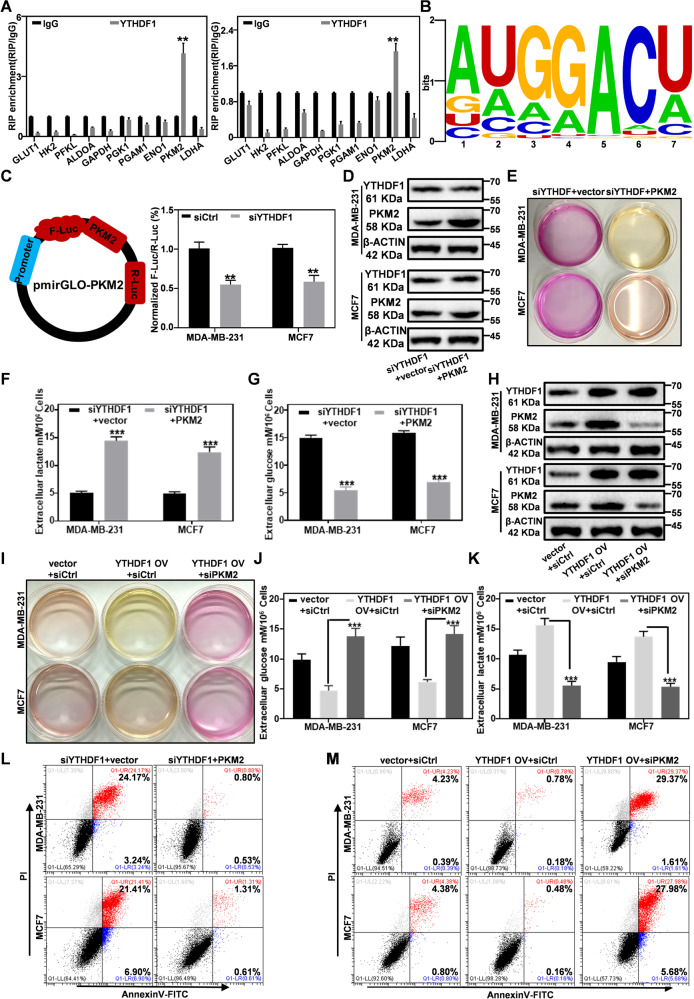
YTHDF1 regulates PKM2 expression to affect glycolysis. **A** RT-PCR analysis on the correlation between YTHDF1 and PKM2 mRNA. **B** The m6A modification motif of PKM2 mRNA for YTHDF1 binding, analyzed by RMBase V2.0 database (http://rna.sysu.edu.cn/rmbase/). **C** Breast cancer cell lines were co-transfected with siYTHDF1 and pmirGLO-PKM2 reporter for 48 h to determine the translation efficiency of PKM2 after different treatment, which was calculated by dividing protein yield with mRNA abundance (F-luc/R-luc). **D** YTHDF1 and PKM2 expression levels in breast cancer cells after the co-transfection with YTHDF1 siRNA and pCDNA3.1-PKM2-3×FLAG. **E** pH changes in the culture medium of breast cancer cells in different groups after 48 h. **F**, **G** The concentration of glucose and lactic acid in the culture medium of the breast cancer cells in different groups after 48 h. **H** Detection of YTHDF1 and PKM2 expression in the breast cancer cells after co-transfection with pCDNA3.1-YTHDF1-3×FLAG and PKM2 siRNA. **I** pH changes in the culture medium of breast cancer cells in different groups after 48 h. **J**, **K** The concentration of glucose and lactate in the culture medium of the breast cancer cells after 48 h. **L** Apoptosis levels of breast cancer cells after co-transfection with YTHDF1 siRNA and pCDNA3.1-PKM2-3×FLAG via FACS. **M** Apoptosis levels of breast cancer cells after co-transfection with pCDNA3.1-YTHDF1-3×FLAG and PKM2 siRNA via FACS. Statistical analysis results are presented as mean ± SEM, student’s t test, *P < 0.05, **P < 0.01, ***P < 0.001.

The hypoxia microenvironment is intrinsically linked to the glycolytic metabolism of breast cancer cells, through which the breast cancer cells would discharge large amount of lactate end-product into the tumor extracellular microenvironment, leading to rapid acidification therein [45–47]. Considering the connection between hypoxia and YTHDF1, we first investigated the changes in lactate levels of the extracellular microenvironment of hypoxic tumors after YTHDF1 inhibition on MDA-MB-231 and MCF7 cells. We found that YTHDF1 depletion evidently ameliorated the acidification of the extracellular microenvironment for hypoxic breast cancer cells, accompanied with significantly reduced glucose consumption and lactate production levels (Supplementary Fig. S5A–D; Original western blot data 10), suggesting that YTHDF1 knockdown inhibited the glycolytic metabolism in breast cancer cells. To further elucidate the role of YTHDF1 in the glycolysis regulation network of breast cancer cells, we employed qPCR to detect the changes in glycolysis-related genes after the RIP treatment with YTHDF1 antibody. The experimental results showed that PKM2 mRNA was efficiently enriched by YTHDF1 (Fig. 5A). As YTHDF1 regulates the expression of target genes by recognizing and binding to m6A modification motifs of mRNA, we searched in RMBase v2.0 database [48] to identify the m6A modification motif of YTHDF1 binding to PKM2 mRNA for determining the interaction between YTHDF1 and PKM2. According to the research data under entry GSE63591 [49], which used RAP-CLIP to identify the YTHDF1-binding m6A motif, the mRNA m6A modification motif of PKM2 for YTHDF1 binding was ATGGACT (Fig. 5B). To further elucidate the PKM2 regulation mechanism of YTHDF1 after recognizing m6A modification sites, we detected the mRNA and protein levels of PKM2 after YTHDF1-knockdown in a time-dependent manner via inhibiting transcription and translation with actinomycin D (ActD) and cycloheximide (CHX), respectively. The experimental results showed that YTHDF1 knockdown has no obvious effect on the cellular abundance and degradation of PKM2 mRNA (Fig. S5F, G). Meanwhile, YTHDF1 knockdown lowered the protein abundance of PKM2 but had no significant effect on the relative content of PKM2 protein over time after CHX-mediated translation inhibition (Fig. S5E, H; Original western blot data 11, 12). The above results indicate that YTHDF1 knockdown has no obvious impact on the stability of both PKM2 mRNA or protein. By referring to the work by Xinyao Lin et al. [22], we speculated that YTHDF1 may affect the protein translation process of PKM2 and therefore constructed the pmirGLO-Snail luciferase reporter gene by ligating the CDS of PKM2 to the multiple cloning sites (MCSs). The dual luciferase assay showed the translation efficiency of PKM2 in siYTHDF1 cells has decreased by about 40% compared to the siCtrl group (Fig. 5C). These observations collectively demonstrated that YTHDF1 affects PKM2 signaling primarily through mediating PKM2 protein translation rather than affecting its mRNA or protein stability.

We further investigated the involvement of YTHDF1-PKM2 axis in breast cancer progression and found that re-expressing PKM2 after YTHDF1 knockout could efficiently restore the glycolysis activity in breast cancer cells (Fig. 5D–G; Original western blot data 8). The simultaneous overexpression of YTHDF1 and knockdown of PKM2 significantly inhibited the glycolysis despite YTHDF1 overexpression (Fig. 5H–K; Original western blot data 9). Cell invasion tests also showed that PKM2 knockdown significantly inhibited the invasiveness of YTHDF1-overexpressing breast cancer cells (Supplementary Fig. S5I–L). In addition, flow cytometry analysis showed that re-expressing PKM2 can reverse the apoptosis induced by YTHDF1 knockdown, while the combined treatment of PKM2 knockdown and YTHDF1 overexpression still led to severe apoptosis of breast cancer cells (Fig. 5L, M). These results indicate that YTHDF1 affects the glycolysis in breast cancer cells by regulating the expression of PKM2, thus imposing a profound impact on the various biological activities of breast cancer cells.

### Down-regulation of YTHDF1 inhibits the growth of breast cancer in vivo

**Fig. 6:**
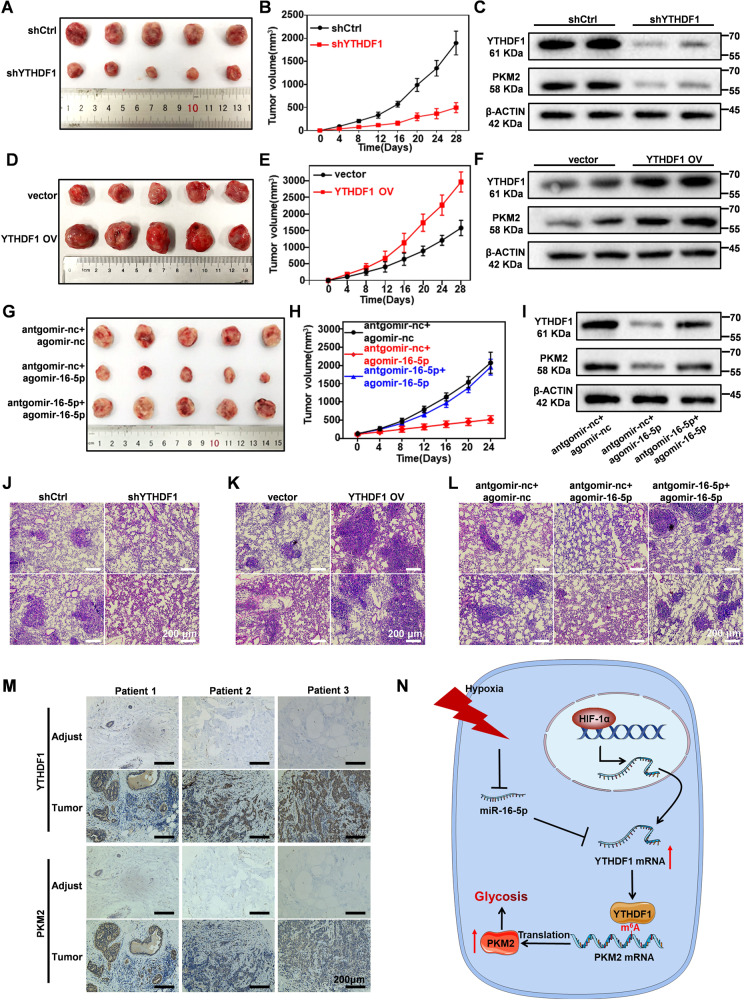
YTHDF1 as a therapeutic target for regulating tumorigenesis and metastasis of breast cancer cells in mice. **A**, **B** Impact of YTHDF1 inhibition on the growth of subcutaneous tumors. **C** Western blot analysis regarding YTHDF1 and PKM2 expression in subcutaneous tumors at 28 days after transplantation. **D**, **E** Impact of YTHDF1 overexpression on the growth of subcutaneous tumors. **F** Western blot analysis regarding YTHDF1 and PKM2 expression in subcutaneous tumors. **G**, **H** Impact of agomir-16-5p transfection on the growth of subcutaneous tumors. **I** Western blot analysis regarding YTHDF1 and PKM2 expression in subcutaneous tumors at 24 days after agomir-16-5p treatment. **J**–**L** Immunohistochemical image regarding the lung metastasis of breast tumors in mice after YTHDF1 knockdown, YTHDF1 overexpression or agomir-16-5p treatment. **M** Immunohistochemical imaging of YTHDF1 and PKM2 in matched normal and cancerous tissues of breast cancer patients. **N** Schematic illustration showing YTHDF1 upregulation mediates hypoxia-dependent breast cancer growth and metastasis through regulating PKM2 to affect glycolysis.

To validate the roles of YTHDF1 in breast cancer progression, we further monitored the effect of YTHDF1 knockdown on the tumorigenicity of breast cancer cells using BALB/c mice with subcutaneous breast tumors, which were established by injecting YTHDF1 shRNA-treated 4T1 cells into 4-week-old female BALB/c mice. We observed that YTHDF1 depletion caused significant reduction in tumor size and increasing apoptosis populations compared to the control group and was accompanied with decreasing PKM2 expression in tumor tissues (Fig. 6A–C and Supplementary Fig. S6A, D; Original western blot data 13), indicating that the YTHDF1 knockdown could effectively inhibit the growth of breast tumors in mice by suppressing PKM2. Moreover, YTHDF1 silencing also substantially reduced lung metastasis of breast tumors in mice (Fig. 6J and Supplementary Fig. S6G). Furthermore, we monitored the effect of YTHDF1 overexpression on the oncogenicity of breast cancer cells in BALB/C mice, which was assessed by monitoring the tumor size changes after injecting YTHDF1 OV transfected 4T1 cells into 4-week-old female BALB/C mice. We found that YTHDF1 overexpression led to a significant increase in the size and PKM2 expression level of subcutaneous breast cancer tissues compared with controls (Fig. 6D–F and Supplementary Fig. S6B, E; Original western blot data 14). Meanwhile, YTHDF1 overexpression also significantly augmented the lung metastatic capacity of breast cancer cells (Fig. 6K and Supplementary Fig. S6H), indicated by the increasing number of metastatic nodules in the YTHDF1 OV-treated group. These observations collectively demonstrated that YTHDF1 is a positive regulator of the oncogenesis and metastasis of breast cancer cells through the downstream PKM2 signaling. Extending from the results above, we evaluated the miR-16-5p-mediated YTHDF1 inhibition and its anti-tumor effect in vivo using similar set-ups. Compared with the negative control (antgomir-nc + agomir-nc), the treatment with miR-16-5p induced a significant decrease in the expression of YTHDF1 and PKM2 in the tumor tissues. Consistent with the trends in YTHDF1 and PKM2 expression, the tumor growth in the agomiR-16-5p treatment group was evidently inhibited, in which the average tumor volume after 24 days of administration was about 1/4 of that of the control group (Fig. 6G–I and Supplementary Fig. S6C, F; Original western blot data 15). The results regarding lung metastasis of the breast tumor in mice also showed that the number of lung metastasis nodes in the miR-16-5p group were much fewer compared to the other two groups (Fig. 6L and Supplementary Fig. S6I). Western blot analysis on the extracted tumor tissues showed similar results with the in vitro tests that the depletion of YTHDF1 inhibited PKM2 expression in xenograft tumors in mice. We further examined the YTHDF1 and PKM2 expression levels in matched pairs of patient-derived breast tumor and adjacent normal tissue using Immunohistochemistry, of which the results showed that both YTHDF1 and PKM2 were upregulated in tumor tissues and supported the positive correlation in between (Fig. 6M). In summary, our results indicate that YTHDF1 plays a key role in promoting breast tumor growth and metastasis in vivo, while miR-16-5p exhibits potential anti-tumor effects by selectively inhibiting YTHDF1.

## Discussion

While m^6^A modification disorder is known to play a significant role in many types of cancer indications [50–52], its involvement in the development and progression of breast cancer is still not well-understood. Herein, our work demonstrates that YTHDF1, a m^6^A-binding protein capable of inducing the translation of the m^6^A-marked mRNAs, is abnormally upregulated in multiple types of breast cancer cell lines and has major roles in driving the genesis, proliferation and metastasis of breast cancer cells. The YTHDF1 upregulation is also intrinsically linked with reduced survival time and poor prognosis for breast cancer patients, suggesting the cancer-driving functions of YTHDF1 thereof. We also used specific siRNAs to silence YTHDF1 in breast cancer cells and observed that YTHDF1 knockdown would significantly inhibit cell proliferation through inducing apoptosis, indicating that YTHDF1 could be exploited as a potential therapeutic target for breast cancer treatment. Our work identified that the abnormal YTHDF1 in breast cancer cells was driven by the hypoxic microenvironment, which could induce the expression of HIF1α while inhibiting endogenous miR-16-5p, of which the latter could interact with 3′UTR of YTHDF1 mRNA to inhibit YTHDF1 expression.

We further investigated the regulatory mechanisms of YTHDF1 on the malignancy of breast cancer cells and confirmed that YTHDF1 was capable of regulating glycolysis in breast cancer cells to exert tumor-driving functions. Studies have shown that YTHDF1 promotes the recruitment of transcription initiation factors such as EIF3 to the mRNA and then initiates protein translation by recognizing and binding to the m^6^A modification site of target gene mRNA [53–55]. Specifically, we found that YTHDF1 had a strong binding ability to the mRNA of PKM2 in breast cancer cell, which is a key enzyme in the glycolysis pathway and responsible for high glycolytic metabolism of YTHDF1-overexpressing tumors. The genetic depletion of YTHDF1 would significantly reduce the PKM2 abundance and inhibit glycolysis in breast cancer cells for effective tumor suppression.

To investigate the therapeutic potential of the YTHDF1-PKM2 axis for the treatment of breast cancers, we tested the antitumor efficacy of miR-16-5p in clinically relevant models. The results showed that the effect of miR-16-5p treatment was similar to that of shRNA-YTHDF1 mediated YTHDF1 silencing effectively abolishing the growth and lung metastasis of subcutaneous breast cancer in mice. The results of immunohistochemical analysis also showed that after miR-16-5p treatment the expression levels of YTHDF1 and PKM2 in tumor tissues were significantly down-regulated, indicating that miR-16-5p can selectively target the YTHDF1-PKM2 signal axis to inhibit tumor growth. Extending from glycolysis-inhibition function of miR-16-5p, we observed that miR-16-5p could also remodel the tumor microenvironment by disrupting the effusion of glycolysis-generated lactic acid, thus conferring an additional benefit on suppressing the growth and metastasis of breast cancer cells while potentiating synergy with other antitumor modalities.

In conclusion, this study confirmed that YTHDF1 is overexpressed in breast cancer and associated with poor prognosis, while hypoxia can promote the expression of YTHDF1 by inducing HIF1α at transcriptional level and inhibiting endogenous miR-16-5p expression at post-transcriptional level. Inhibiting YTHDF1 would suppress the expression of the glycolytic gene PKM2 and impair the glycolytic activity of breast cancer cells, eventually leading to reduced proliferation and metastasis potential. In this study, miR-16-5p acts as a tumor suppressor by selectively inhibiting YTHDF1, which could be exploited as a potential therapy for breast cancer. The results are consistent with functional roles of miR-16-5p as a tumor suppressor and chemosensitizer reported by Hongliang Zhang et al. and Huiping Li et al. [56, 57]. Overall, our findings on the breast cancer-driving functions of YTHDF1 could improve our understanding on the involvement of m^6^A modification in the occurrence of cancer and also provides a strong rationale for the therapeutic targeting and prognostic evaluation of YTHDF1 in various breast cancer indications.

## Materials and methods

### Handling of human breast cancer samples from real-life patients

The breast cancer and normal breast tissues were provided by the Cancer Hospital of Chongqing University and Xinqiao Hospital of Army Medical University. The study was conducted in compliance with to the ethical guidelines related to the research of human participants. For fresh breast tumors and adjacent normal tissues, they were dissected during surgery and then immediately transferred to RNAlater solution for storage. The paraffin-embedded specimens were collected from the Cancer Hospital of Chongqing University. Breast cancer cell lines MDA-MB-231 (#CRL-12532), MCF 7 (#HTB-22), T-47D (#HTB-133), BT-474 (#HTB-20) and normal breast cell line MCF 10A (#CRL-10317) were purchased from American Type Culture Collection (ATCC) with authentication. These cell lines were cultured in Dulbecco’s modified Eagle’s medium (DMEM, Corning, USA) containing 10% fetal bovine serum and 1% penicillin/streptomycin solution. Cells were grown in a constant temperature incubator at 37 °C with 5% CO_2_.

### siRNA or microRNA transfection

Interferential RNAs or microRNAs were transfected into breast cancer cells with Lipofectamine 3000 (Thermofisher, L3000015). When the cell confluence reached 60–80%, different siRNAs or microRNAs were transfected. Similarly, when the cell confluency reached 60–80%, different siRNA or microRNA (200 ng) and the constructed expression plasmid (1 μg) were co-transfected into breast cancer cells. All short RNAs and microRNAs were synthesized by Suzhou Beixin Biotechnology Co., Ltd (siYTHDF1 1: LZ0501. 200 ng; si YTHDF1 2: LZ0502. 200 ng; miR-16-5p mimics: LZ0503. 200 ng; miR-16-5p inhibitor: LZ0504. 200 ng).

### Construction and transfection of plasmid

The plasmid for shYTHDF1 was constructed based on the pLKO.1 vector (Addgene, #8453) with the shYTHDF1 sequence (5′-GGACATTGGTACTTGGGATAA-3′), while mouse YTHDF1 overexpression plasmid was prepared by inserting YTHDF1 (NM_173761.3) into pLVX- puro vector (Addgene, #125839). Human YTHDF1 and PKM2 overexpression vectors were constructed by inserting YTHDF1 (NM_017798.4) and PKM2 (NM_002654.6) into pCDNA3.1-3×FLAG (Addgene, #53556) plasmid respectively, of which the sequences were synthesized by Miaolingbio Ptd Ltd.

The as-prepared plasmids (1 μg) were transfected individually in HEK-293T together with 1 μg of packing constructs pMD2.G (Addgene, #12259) and pSPAX2 (Addgene, #12260). Then the cultured supernatant containing the lentivirus was collected and filtered with a sterile filter membrane with a diameter of 0.45 μm. 500 μL of the filtered lentivirus mixture was then diluted with culture medium to a volume of 1 mL and further transfected into the breast cancer with Lipo3000 (Thermo). Breast cancer cell lines with YTHDF1 knockdown or overexpression were screened with puromycin (1 mg/mL).

### Immunohistochemical analysis

For the immunohistochemical analysis of the clinical samples, fresh breast cancer tissue specimens were fixed with 4% paraformaldehyde and embedded in paraffin, and then cut into thin slices. The tissue sections were subsequently deparaffinized with xylene and rehydrated with ethanol, and the endogenous peroxidase was blocked with 4% H_2_O_2_. Afterwards, the tissue sections were boiled in a steamer with antigen retrieval solution for 20 min and further treated with Quickblock for 30 min. Primary antibodies of anti-YTHDF1 (Proteintech, #17479-1-AP) and anti-PKM2 (Abcam, #ab85542) were later added for incubation overnight at 4 °C. The samples were washed and incubated with peroxidase-labeled secondary antibodies and 3,30 diaminobenzidine, and eventually counterstained with hematoxylin for microscopic examination.

### Cell proliferation and apoptosis assays

For CCK8 analysis, 8000 cells were seeded into each well of a 96-well plate with fresh culture medium. Cell Counting Kit-8 (Solarbio, # CA1210) was used to detect cell viability after 48 h of transfection treatment, and the microplates were incubated at 37 °C. After 4 h, absorbance was recorded at 450 nm using a microplate reader (ThermoFisher) and the relative viability calculated using untreated cells as control (100%). For EdU (5-Ethynyl-2′-deoxyuridine) assay, EdU (Beyotime, # C0071S) was added to the six-well plate and incubated the cells in the logarithmic growth phase for 3 h according to the provided instruction. The cells were washed twice with PBS for 5 min, and then fixed with 4% paraformaldehyde for 30 min. The cells were washed twice with PBS for 5 min each time and infiltrated with 0.3% TritonX-100 in PBS before being stained with the reaction solution. The images were captured with a Leica laser confocal microscope. For the apoptosis assay, cells were incubated staining using Annexin V-PI Apoptosis Detection Kit (Beyotime, #C1065M) according to the provided instruction, followed by flow cytometry analysis (Beckman).

### Western blot analysis

The cell samples were lysed by cell lysate buffer (Beyotime, P0013) and later centrifuged to collect the supernatant, which was boiled for ten minutes to obtain the protein samples, which were then electrophoresed and transferred to polyvinylidene fluoride (PVDF) membrane (Millipore, ISEQ000010). The transfer membranes were blocked in 5% non-fat milk at room temperature for 1 h, and then incubated with the primary antibodies of YTHDF1 (Proteintech, #17479-1-AP), PKM2 (Abcam, #ab85542), HIF1α (ABclonal, #A17906), HIF2α (Abcam, #ab109616) and β-actin (ABclonal, #AC004) at 4 °C overnight, following the standard procedures from manufacturer’s instructions. Afterward the samples were incubated with secondary antibodies and washed three times. Finally, the electrochemiluminescence (Solarbio, # PE0010) signal was captured on the gel imaging system (BIO-RAD).

### RNA extraction and RT-PCR analysis

The total RNA was isolated with Trizol (Invitrogen) according to the instructions provided by the manufacturer, and the quality of RNA was analyzed by a spectrophotometer. Complementary DNA was synthesized using SuperScript IV CellsDirect cDNA kit (Invitrogen) according to the instructions provided. Real-time quantitative fluorescent PCR was performed on real-time PCR (BIO-RED, CFX384) with SYBR Green Master mix (Takara), and β-actin was used as an internal standardization control to calculate relative mRNA expression level.

### RNA immunoprecipitation

RNA immunoprecipitation was performed with the RNA immunoprecipitation (RIP) kit (BersinBio, #Bes-5101-1) according to the instruction provided by the manufacturer. In brief, the magnetic bead suspension containing 5 μg of anti-mouse immunoglobulin G (BersinBio, #Bes-5101-1) or anti-YTHDF1 (Proteintech, #17479-1-AP) antibodies was incubated with pre-cooled cell lysate at 4 °C overnight. The associated RNA-protein complexes were collected and washed three times, followed by proteinase K digestion and RNA isolation with TRIzol. The interaction between protein and RNA was analyzed by RT-PCR and normalized by Input.

### Luciferase reporter assays

The YTHDF1 3′ UTR wild-type and mutant-type dual-luciferase vectors were synthesized by Miaolingbio Ptd Ltd. The luciferase activity is measured by a dual luciferase reporter gene detection kit (ThermoFisher, # 16186) in a fluorescence microscope (Ax to vert ZooM Carl Zeiss). And then the firefly luciferase activity values were normalized to the Renilla luciferase activity values that reflect expression efficiency.

### Transwell invasion assay and clonogenic assays

For transwell invasion assay, 1.5 × 10^5^ cells were seeded on Matrigel in a chamber (Corning) and mixed homogenously with serum-free medium. At the same time, the serum-supplemented medium was added to a 24-well culture plate. Then, cells were collected and washed three times with PBS, and then fixed with 4% paraformaldehyde for 30 min. The fixed samples were washed three times with PBS, and finally stained with crystal violet staining solution (Beyotime, #C0121) for 30 min and imaged on a microscope (Nikon).

For the clonogenic assay, 500 cells were seeded onto a 60 mm^2^ cell culture dish containing fresh 10% bovine serum medium and continue culturing for 2 weeks until the colonies became visible to the naked eye. Then samples were stained with crystal violet staining solution for 30 min, and photographed with a microscope to collect images.

### Determination of glucose and lactate concentrations

The supernatant of culture medium after 48 h of cell cultured was collected and assayed by glucose assay reagent (Sigma #3091314) or lactate assay kit (Solarbio #BC2235) to detect the glucose consumption and lactate production of the cells, respectively. The concentration of glucose or lactate in each sample was calculated via standard curve calibration.

### RNA stability

To measure the mRNA stability of PKM2 in cells after YTHDF1 knockdown, we treated cells with 5 μg/mL actinomycin D (ActD, Sigma), harvested cells at indicated time points, and isolated RNA for detection by RT-PCR.

### Protein stability

To determine the protein stability of PKM2 after YTHDF1 knockdown, we treated breast cancer cells with 100 μg/mL cycloheximide (CHX, Sigma), collected cells at the indicated time points, and extracted proteins for western blot analysis.

### Protein translation

Based on a previous report [22], we constructed a pmirGLO-PKM2 luciferase reporter gene vector by ligating the CDS of PKM2 to a MCS, followed by dual luciferase assay after co-transfection of YTHDF1 siRNA with pmirGLO-PKM2.

### TCGA data analysis

The expression of YTHDF1 and overall survival (OS) was retrieved from the data of The Cancer Genome Atlas (TCGA) (https://cancergenome.nih.gov/). The transcripts per million (TPM) of YTHDF1 in normal or cancer tissues was Log transformed (Log2), and significance of difference was analyzed by Student’s t test. The OS was analyzed by Kaplan–Meier curve in Gene Expression Profiling Interactive Analysis 2 (GEPIA2) webserver (http://gepia2.cancer-pku.cn/#index). The Cutoff-High and Cutoff-Low were both set at 50%.

### Animal study

All animal experiments were carried out on 4–6-week-old female BALB/c mice that were purchased from the Animal Experiment Center of Chongqing Medical University, and all operations were carried out in strict accordance with the Animal Management Rules of the Ministry of Health of the People’s Republic of China (Document No. 55, 2001).

To investigate the effect of YTHDF1 on breast cancer progression in mice, the reared mice were randomly divided into two groups (*n* = 5). Each mouse was injected subcutaneously with 5 × 10^6^ units of tumor cells to construct the tumor model. In the shCtrl group, shCtrl-transfected 4T1 (ATCC, #CRL-2539) cells were injected subcutaneously into mice to construct YTHDF1-normal 4T1 tumors. In the shYTHDF1 group, the shYTHDF1-transfected 4T1 cells were injected subcutaneously into mice to construct the YTHDF1-knockdown 4T1 tumors.

To investigate the effect of miR-16-5p on breast cancer progression by regulating the expression of YTHDF1, three experimental groups (*n* = 5) were designed for the in vivo experiment, which were the agomiR-nc+antgomiR-nc, agomiR-16-5p + antgomiR-nc, and agomiR-16-5p + antgomiR-16-5p groups. When the tumor volume reached 0.5 mm^3^, the xenograft tumor model mice were randomly divided into three groups, the corresponding drugs were administered periodically once every two days by s.c local multipoint injection, and the tumor volume was measured after each injection, calculated using the formula Volume = (L*W^2^)/2. After 3 weeks of treatment, the mice were sacrificed, the tumors were taken out, fixed with 4% paraformaldehyde for 48 h, transferred to 70% ethanol, embedded in paraffin and sectioned. All sections were examined by histological analysis.

## Supplementary information

Supplementary materials

Original data for western blot assays

Reproducibility checklist

## Data Availability

All data generated or analyzed during this study are included in this published article and its supplementary information files.
