# Supplementary material for: YTHDF1 upregulation mediates hypoxia-dependent breast cancer growth and metastasis through regulating PKM2 to affect glycolysis
Source: Cell Death Dis. 2022 Mar 23;13(3):258. doi: 10.1038/s41419-022-04711-1 (PMC8940925; doi:10.1038/s41419-022-04711-1)
Supplement: Supplementary file 1 — Supplementary materials [file 41419_2022_4711_MOESM1_ESM.docx]

**Supplementary Data**

**YTHDF1 upregulation mediates hypoxia-dependent breast cancer growth and metastasis through regulating PKM2 to affect glycolysis**

Xuemei Yao^1†^, Wei Li^2†^, Liqi Li^3*^, Menghuan Li^1^, Youbo Zhao^4^, De Fang^1^, Xiaohua Zeng^2*^, Zhong Luo^1*^

^1^School of Life Science, Chongqing University, Chongqing, 400044, China.

^2^Chongqing Key Laboratory of Translational Research for Cancer Metastasis and Individualized Treatment, Chongqing University Cancer Hospital, Chongqing, 400030, China.

^3^Department of General Surgery, Xinqiao Hospital, Army Medical University, Chongqing, 400037, China.

^4^Center for Tissue Engineering and Stem Cell Research, National Joint Local Engineering Laboratory for Cell Engineering and Biomedicine Technique, Guizhou Medical University, Guiyang, 550004, China.

^†^These authors contributed equally to this work.

Corresponding author:

Dr. Xiaohua Zeng: qq-zxh@126.com

Dr. Liqi Li: liliqi198610@163.com

Prof. Zhong Luo: [luozhong918@cqu.edu.cn](mailto:luozhong918@cqu.edu.cn)


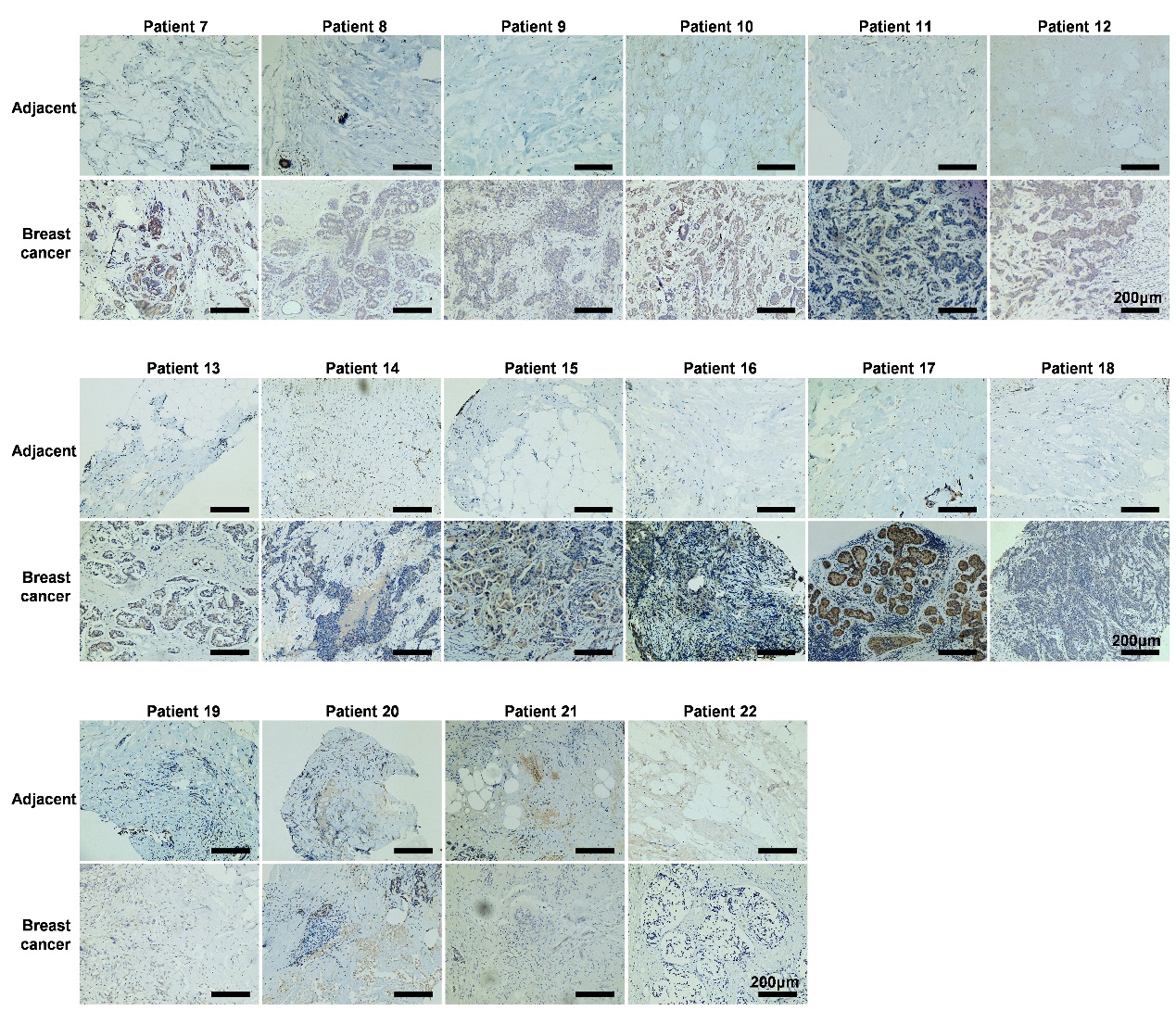


**Figure S1.** Representative immunohistochemical images of YTHDF1 in patient-derived breast cancer and normal breast tissues.


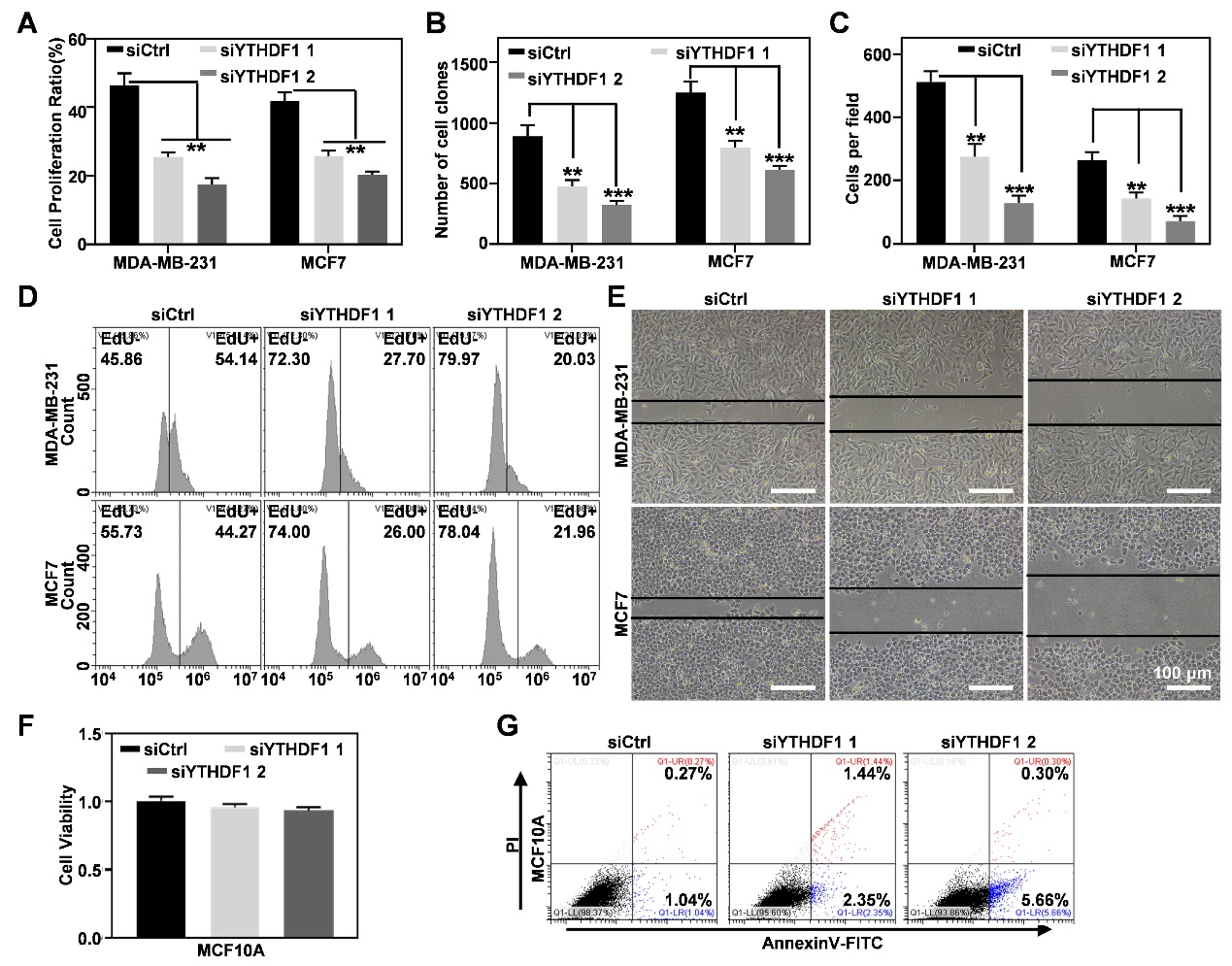


**Figure S2.** **Knockdown of YTHDF1 inhibits cell growth and migration in vitro.** (A, B, and C) Statistical analysis of EdU staining, clone formation and cell invasion results, respectively**.** Data is shown as mean ± SEM. (D) EdU assays of MDA-MB-231 and MCF7 cells after YTHDF1 knock-down by FACS. (E) Knock-down of YTHDF1 decreased the abilities of migration of MDA-MB-231 and MCF7 cells. (F) CCK8 assays on the cell viability of MCF10A cells after siYTHDF1 transfection. (G) FACS analysis on the apoptosis of MCF10A after siYTHDF1 transfection. Statistical analysis results are presented as mean ± SEM (n=3), student’s t test, *P <0.05, ** P <0.01, ***P<0.001.


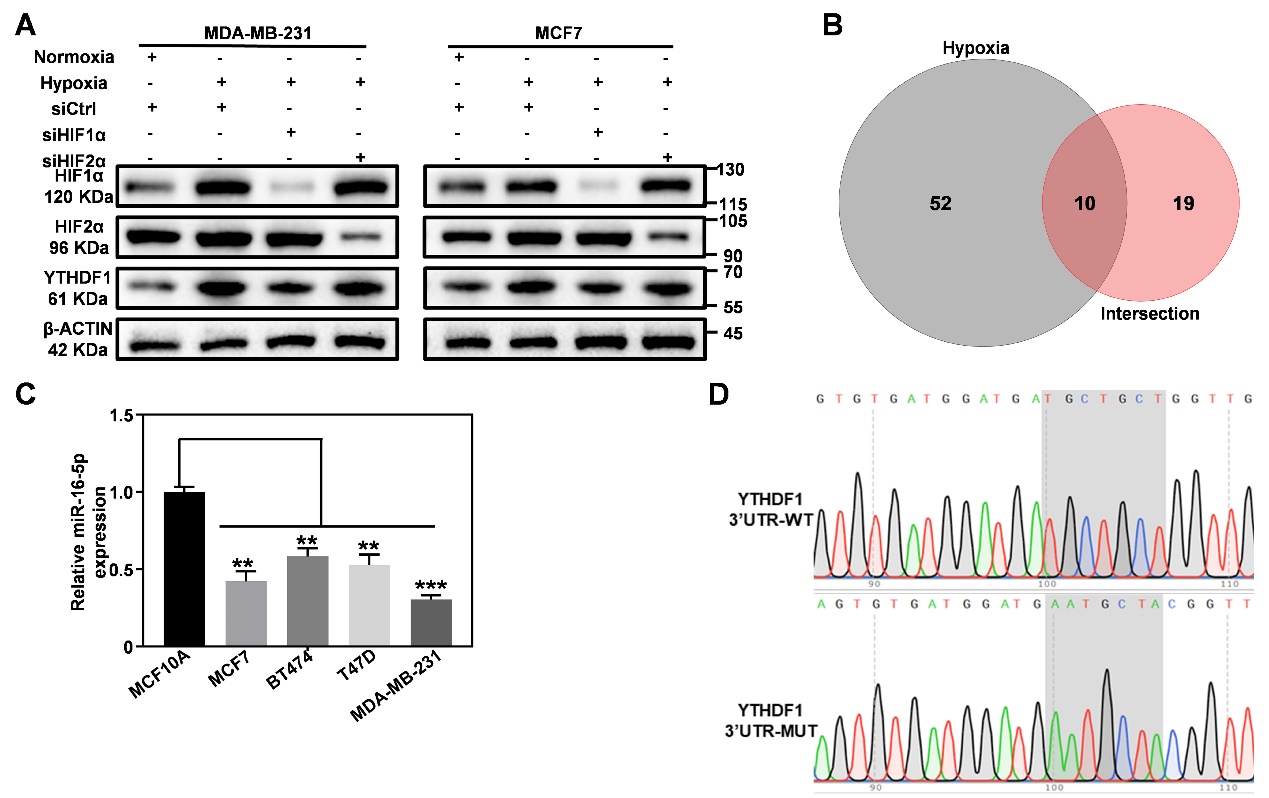


**Figure S3. The mechanism of hypoxia-induced YTHDF1 expression.** (A) Western blot analysis of YTHDF1 expression after knocking down HIF1α and HIF2α under hypoxia induction. (B) Screening of the hypoxia responsive YTHDF1-targeting microRNA in the microRNA database. (C) RT-PCR analysis on the expression levels of miR-16-5p in normal breast and breast cancer cells. (D) Sequencing of wild-type (WT) and mutant (MUT) dual luciferase vectors of the constructed psiCHECK 2-YTHDF1 3'UTR. Statistical analysis results are presented as mean ± SEM (n=3), student’s t test, *P <0.05, ** P <0.01, ***P<0.001.


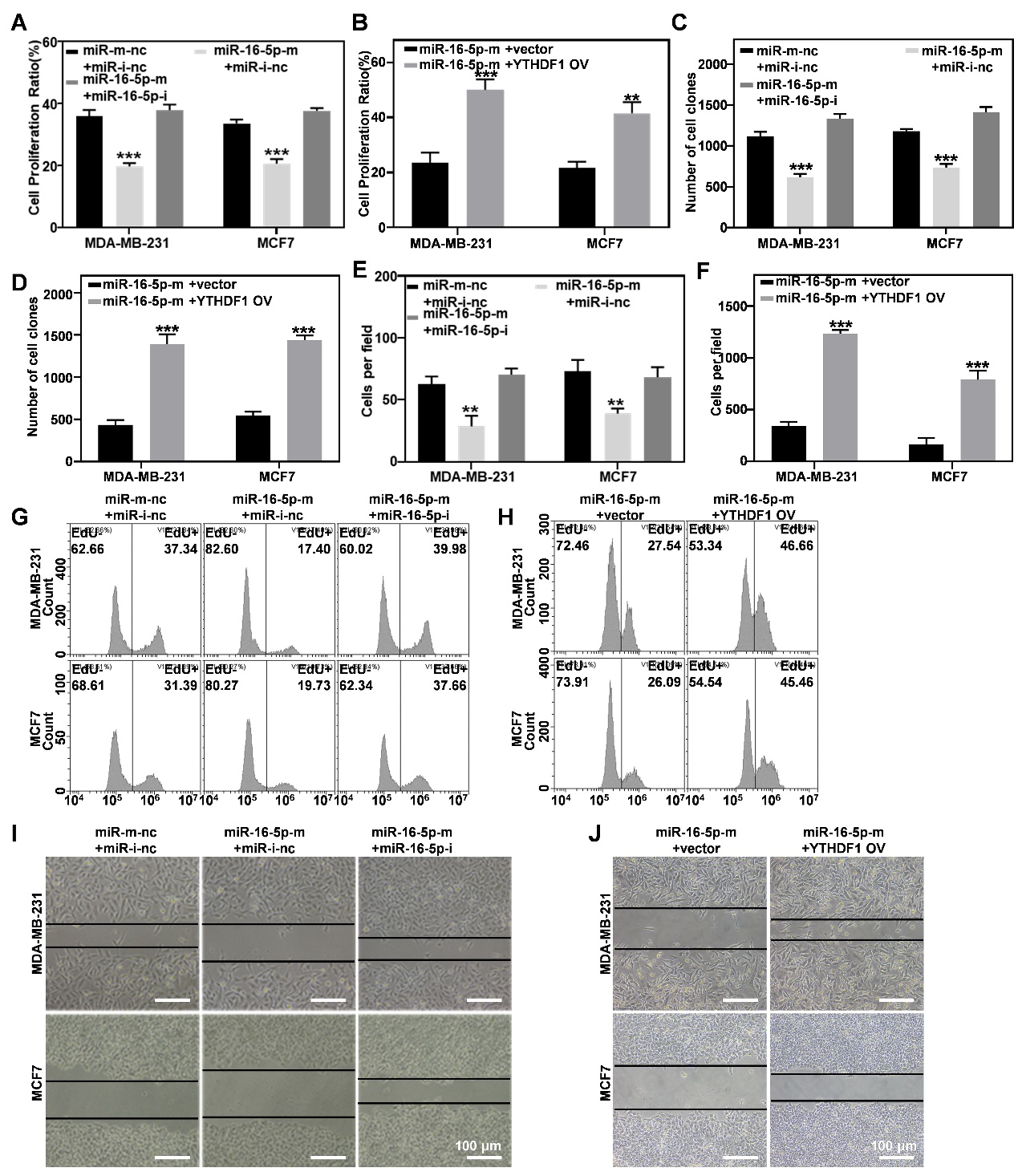


**Figure S4.** **Transfection of miR-16-5p inhibited cell growth and migration in vitro.** (A-F) statistical analysis of EdU staining, clone formation and cell invasion, respectively. Data are shown as mean ± SEM. (G and H) EdU assays of MDA-MB-231 and MCF7 cells after miR-16-5p mimics with/without miR16-5p inhibitors or miR-16-5p mimics with/without pCDNA3.1-YTHDF1-3×FLAG transfection by FACS. (I and J) miR-16-5p inhibited the migration abilities of MDA-MB-231 and MCF7 cells. Statistical analysis results are presented as mean ± SEM (n=3), student’s t test, *P <0.05, ** P <0.01, ***P<0.001.


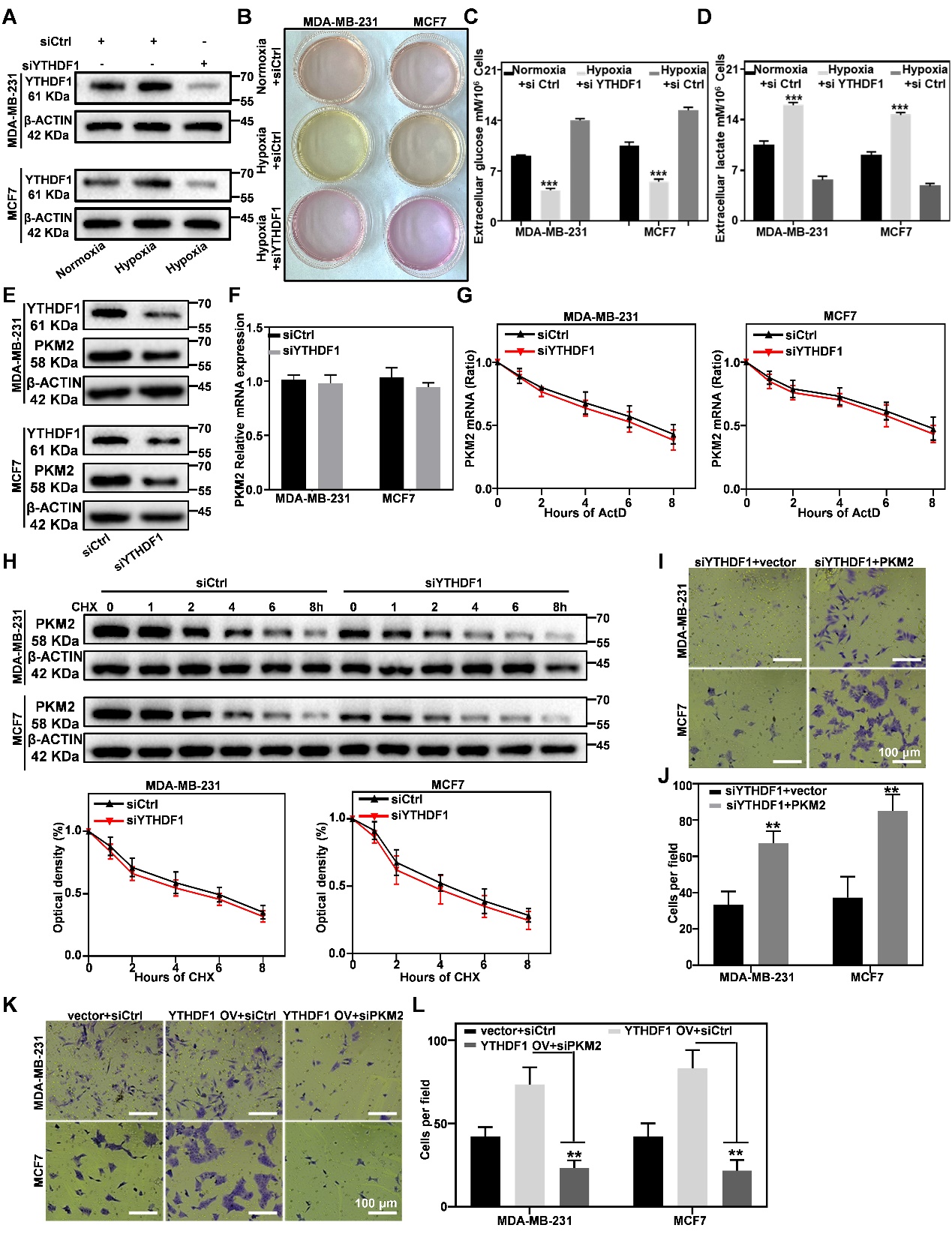


**Figure S5. YTHDF1 regulates glycolysis by regulating the expression of PKM2.** (A) YTHDF1 expression in breast cancer cells after different treatment. (B) pH changes in the tumor extracellular microenvironment after various treatment. (C and D) The concentrations of glucose and lactic acid in the culture medium of the breast cancer cells after 48 hours of different treatment. (E) Western Blot analysis of YTHDF1 and PKM2 expression after knock-down of YTHDF1. (F) Detection of PKM2 mRNA by qPCR after YTHDF1 knock-down. (G) ActD assay on the decay rate of PKM2 mRNA after YTHDF1 knock-down. (H) CHX chase assay on the protein stability of PKM2 after YTHDF1 knock-down. (I and J) Invasion capability of MDA-MB-231 and MCF7 cells after co-transfection with YTHDF1 siRNA and pCDNA3.1-PKM2-3×FLAG. (K and L) Invasion capability of MDA-MB-231 and MCF7 cells after co-transfection with pCDNA3.1-YTHDF1-3×FLAG and PKM2 siRNA. Statistical analysis results are presented as mean ± SEM (n=3), student’s t test, *P <0.05, ** P <0.01, ***P<0.001.


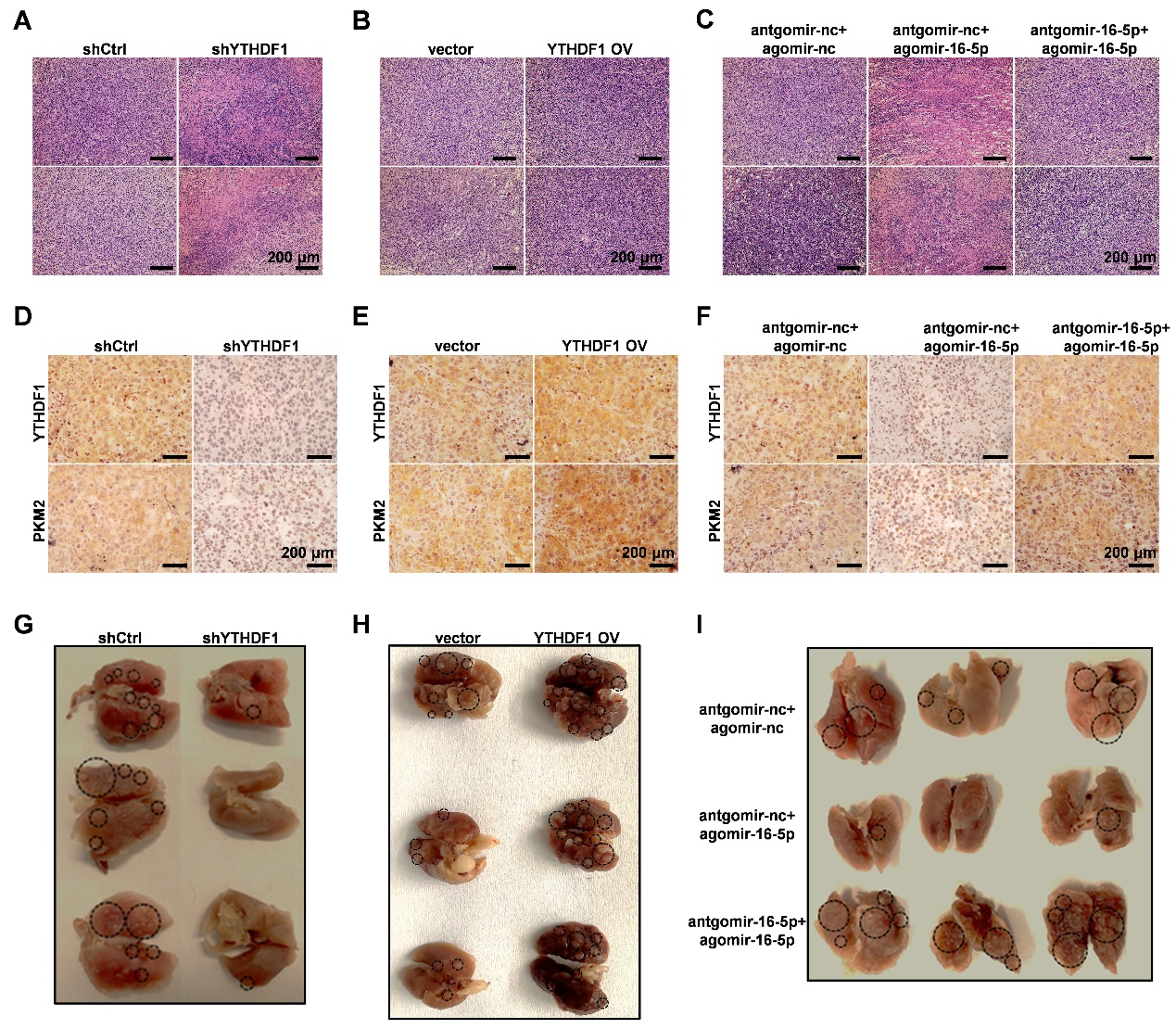


**Figure S6.** **Down-regulation of YTHDF1 inhibits tumorigenicity and metastasis of breast cancer cells.** (A, B and C) Immunohistochemical analysis on the apoptosis of subcutaneous tumor cells after down-regulation/overexpression of YTHDF1. (D, E and F) Immunohistochemical analysis of the expression of YTHDF1 and PKM2 after different treatment in vivo. (G, H and I) Lung metastasis of the breast cancer cells in vivo after YTHDF1 knockdown/overexpression or agomir-16-5p treatment.
