# Supplementary material for: YTHDF1 upregulation mediates hypoxia-dependent breast cancer growth and metastasis through regulating PKM2 to affect glycolysis
Source: Cell Death Dis. 2022 Mar 23;13(3):258. doi: 10.1038/s41419-022-04711-1 (PMC8940925; doi:10.1038/s41419-022-04711-1)
Supplement: Supplementary file 2 — Original data for western blot assays [file 41419_2022_4711_MOESM2_ESM.docx]

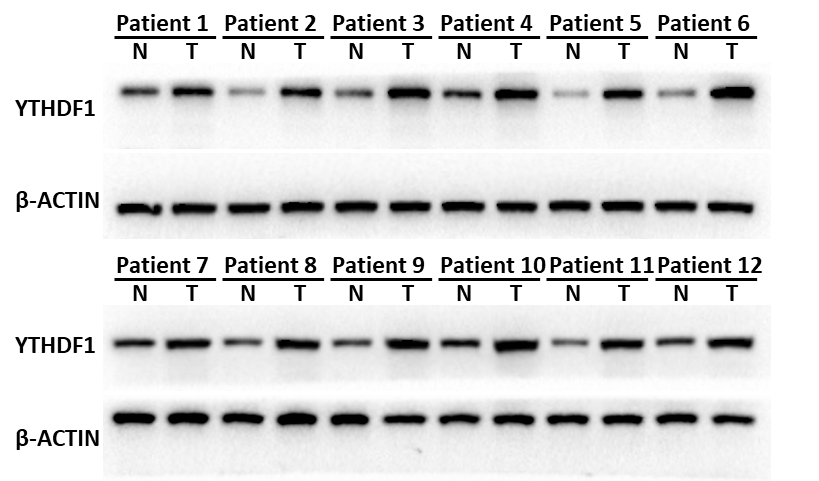


**Original western blot data 1.** Related to Figure 1F.

**
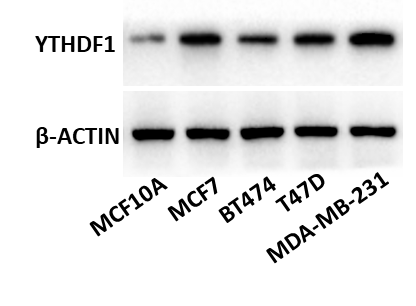
**

**Original western blot data 2.** Related to Figure 1H.

**
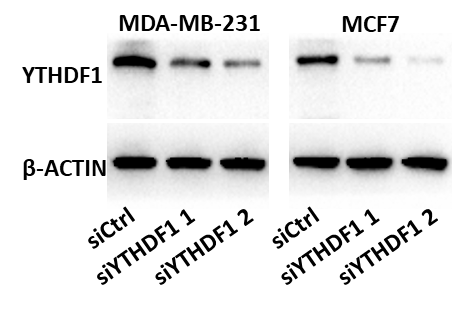
**

**Original western blot data 3.** Related to Figure 2A.

**
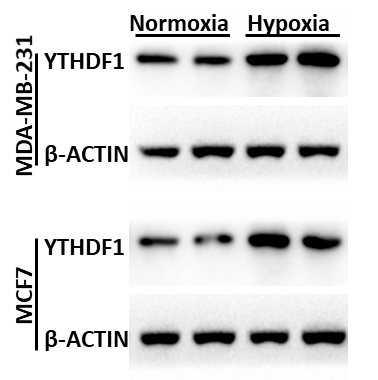
**

**Original western blot data 4.** Related to Figure 3B.

**
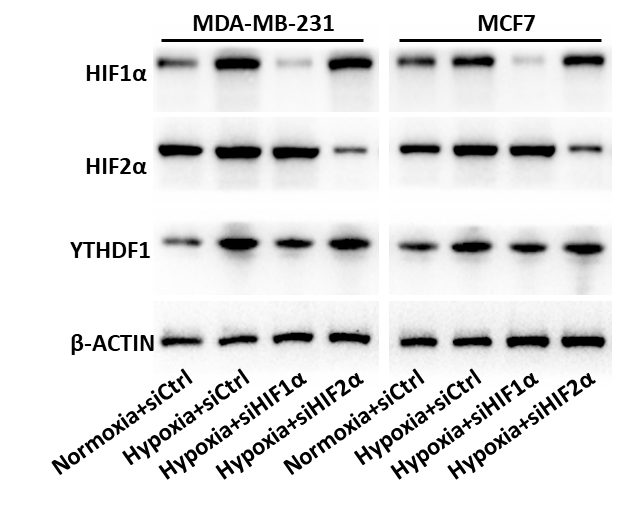
**

**Original western blot data 5.** Related to Figure S3A.

**
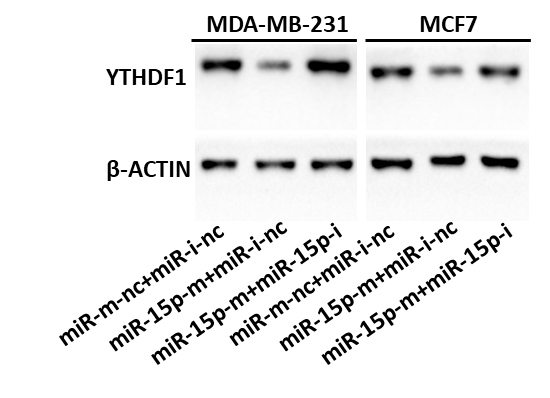
**

**Original western blot data 6.** Related to Figure 4A.

**
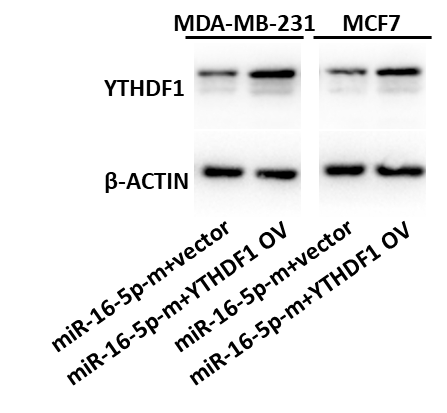
**

**Original western blot data 7.** Related to Figure 4B.

**
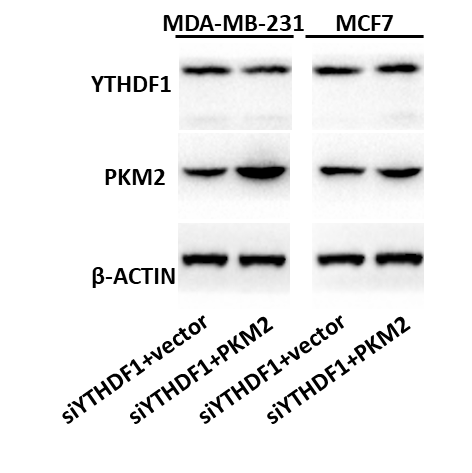
**

**Original western blot data 8.** Related to Figure 5D.

**
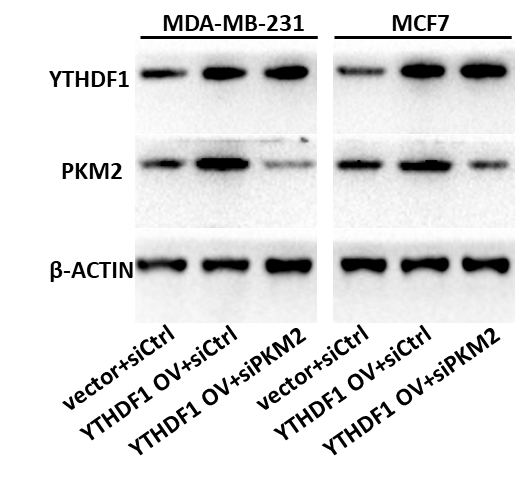
**

**Original western blot data 9.** Related to Figure 5H.

**
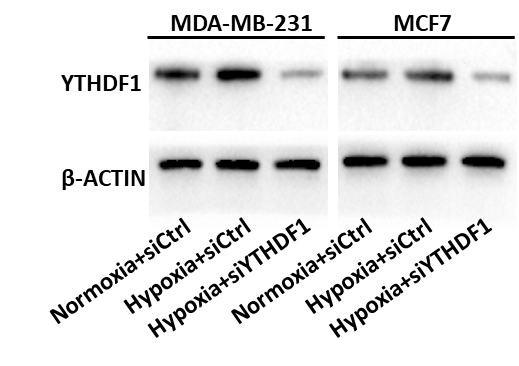
**

**Original western blot data 10.** Related to Figure S5A.

**
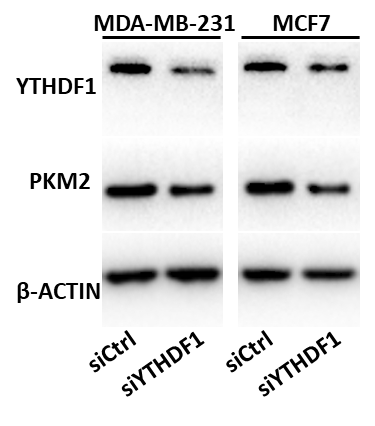
**

**Original western blot data 11.** Related to Figure S5E.

**
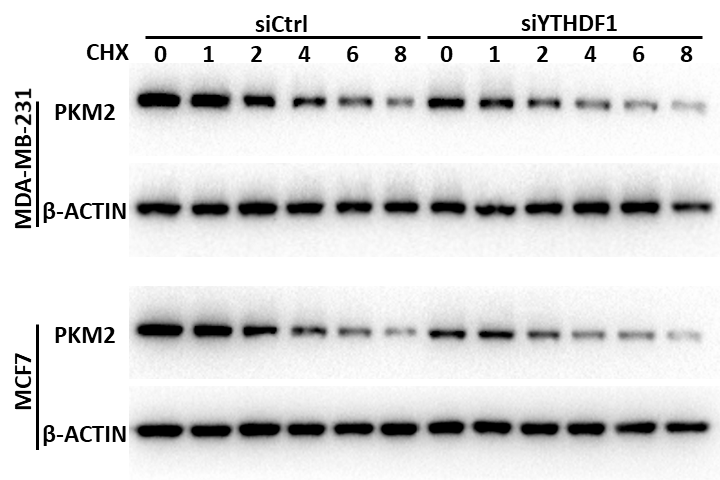
**

**Original western blot data 12.** Related to Figure S5H.

**
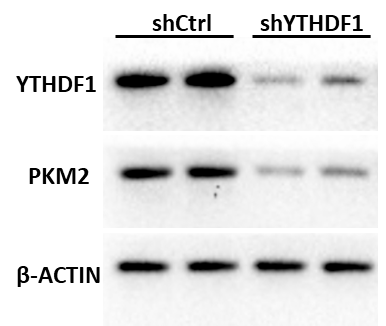
**

**Original western blot data 13.** Related to Figure 6C.

**
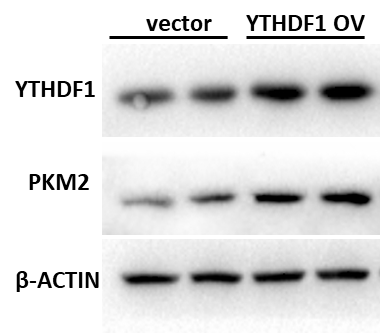
**

**Original western blot data 14.** Related to Figure 6F.

**
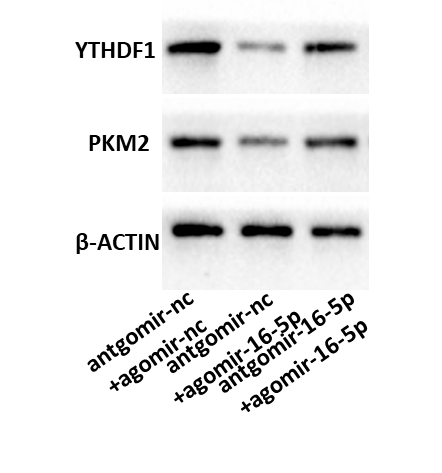
**

**Original western blot data 15.** Related to Figure 6I.
